# Breast-Conserving Therapy for Multiple Ipsilateral Breast Cancer After Neoadjuvant Systemic Therapy

**DOI:** 10.1245/s10434-025-18939-6

**Published:** 2026-01-03

**Authors:** Monika K. Masanam, Jennifer R. Bellon, José P. Leone, Elizabeth A. Mittendorf, Tari A. King, Olga Kantor

**Affiliations:** 1 Division of Breast Surgery, Department of Surgery, Brigham and Women’s Hospital, Dana-Farber Cancer Institute, Boston, MA USA; 2 Breast Oncology Program, Dana-Farber Brigham Cancer Center, Boston, MA USA; 3 Harvard Medical School, Boston, MA USA; 4 Radiation Oncology, Brigham and Women’s Hospital, Boston, MA USA; 5 Medical Oncology, Dana-Farber Cancer Institute, Boston, MA USA; 6 Present Address: Division of Breast Surgery, Department of Surgery, Beth Israel Deaconess Medical Center, Boston, MA USA; 7 Present Address: Division of Breast Surgery, Department of Surgery, Emory University School of Medicine, Atlanta, GA USA; 8 Present Address: Division of Breast Surgery, Department of Surgery, Northwestern University, Chicago, IL USA

**Keywords:** Multiple ipsilateral breast cancer, Neoadjuvant systemic therapy, Breast-conserving surgery

## Abstract

**Background:**

The ACOSOG Z11102 trial demonstrated the safety of breast-conserving surgery (BCS) with adjuvant radiation in women with multiple ipsilateral breast cancer (MIBC) undergoing upfront surgery, reporting a 5-year local recurrence (LR) of ~3%. However, the oncologic safety of BCS in women with MIBC receiving neoadjuvant systemic therapy (NST) remains uncertain.

**Patients and Methods:**

Patients with stage I–III unifocal or MIBC who underwent BCS following NST from 2016 to 2023 were identified in a prospectively maintained institutional database. MIBC was defined preoperatively as the presence of 2–3 foci of biopsy-proven breast cancer with at least 2 cm of intervening normal breast tissue and at least one focus of invasive disease.

**Results:**

A total of 1515 patients were identified: 73 (4.8%) with MIBC and 1442 (95.2%) with unifocal disease. Baseline clinicopathologic characteristics were similar between groups. Median age was 55 years, and most received neoadjuvant chemotherapy (82.2% vs. 83.4%). Molecular subtype distribution was similar between cohorts (*p* = 0.97). Of the patients with MIBC, 48 (65.8%) underwent single-site lumpectomy, 23 (31.5%) two-site lumpectomy, and 2 (2.7%) three-site lumpectomy. At median follow-up of 34.7 months, there was no difference in LR (1.4% vs. 3.1%, *p* = 0.40), distant recurrence (5.5% vs. 4.6%, *p* = 0.37), or breast cancer mortality (4.1% vs. 2.6%, *p* = 0.45) between groups.

**Conclusions:**

In this retrospective analysis of women with MIBC who underwent BCS after NST, local recurrence (LR) was 1.4% at 3-year median follow-up, which was similar to patients with unifocal breast cancer. These findings suggest BCS is a safe surgical option in well-selected patients with MIBC undergoing NST.

**Supplementary Information:**

The online version contains supplementary material available at 10.1245/s10434-025-18939-6.

Breast-conserving surgery (BCS) is the preferred treatment option for patients with unifocal breast cancer with proven oncologic safety and improved cosmetic and psychosocial outcomes.^1–3^ Historically, mastectomy was the recommended surgical approach for patients with multiple ipsilateral breast cancer (MIBC). This recommendation was based on several small retrospective studies reporting higher rates of local recurrence (LR) following BCS when compared with patients with unifocal disease.^4–6^ Advancements in breast cancer diagnosis and treatment such as enhanced imaging modalities and more effective, tailored systemic therapies have since led to improvements in outcomes for patients with MIBC, including better local control.^7^

The prospective single-arm phase II American College of Surgeons Oncology Group (ACOSOG) Z11102 clinical trial was conducted to assess LR rates in patients with MIBC undergoing upfront BCS and radiation therapy (RT).^8^ MIBC was defined as two or three foci of biopsy-proven breast cancer with at least 2 cm of intervening normal tissue and at least one site of invasive disease, allowing ductal carcinoma in situ (DCIS) or invasive breast cancer as the additional sites. Overall, 270 patients were enrolled from 2012 to 2016 with a primary endpoint of LR. Estimated 5-year cumulative rate of LR was 3.1%, which was below the predefined clinically acceptable threshold of 8%. Cosmetic outcome, a planned secondary endpoint, was also favorable with 70.6% of women reporting good or excellent cosmesis on a four-point patient-reported survey.

While ACOSOG Z11102 has set the stage for BCS and RT as a safe and effective option for MIBC in the upfront surgery setting, patients who received neoadjuvant systemic therapy (NST) were excluded from the trial and the safety of this approach after NST is uncertain. In this study, we compare clinicopathologic characteristics and oncologic outcomes between patients with unifocal breast cancer and MIBC selected for BCS after NST at our institution.

## Patients and Methods

A prospectively maintained database that is approved by our Institutional Review Board was used to identify patients aged 18 or older diagnosed with clinical stage I–III invasive breast cancer and treated with NST followed by BCS between 2016 and 2023. Case selection for unifocal breast cancer and MIBC was based on pretreatment imaging. Unifocal breast cancer was defined as a single biopsy-proven primary tumor. MIBC was defined as in the ACOSOG Z11102 trial as the presence of two or more distinct biopsy-proven foci of carcinoma within the same breast, with at least 2 cm of intervening normal tissue, confirmed on imaging and histopathology.^8^ At least one tumor focus had to be invasive, and DCIS was allowed for additional foci of disease. All patients received NST, consisting of either neoadjuvant chemotherapy (NAC) or neoadjuvant endocrine therapy (NET). BCS for MIBC was performed through a single- or multiple-site lumpectomy, determined by the operative and pathology report descriptions. Single-site lumpectomy was defined as taking all sites of disease out in one lumpectomy specimen, while a multiple site lumpectomy was defined as taking the sites of disease out in at least two separate specimens. The approach taken was at the discretion of the surgeon. Patients were excluded if they had less than 2 cm distance between tumor foci, contiguous intervening disease on imaging, or bilateral breast cancer. For histologic grade, the highest grade among the foci was reported. MIBC was characterized as invasive ductal carcinoma (IDC), invasive lobular carcinoma (ILC), or mixed if features of both invasive ductal and lobular carcinoma were present. Margins were routinely taken as cavity shaves for each lumpectomy site, and considered negative if there was no tumor on ink. Of note, magnetic resonance imaging (MRI) was clinician-dependent and not routinely utilized for all NST patients.

The primary outcome of interest was LR, defined as histologically confirmed invasive breast cancer or DCIS in the ipsilateral breast after completion of prior BCS. Secondary outcomes were any distant recurrence (DR) and breast-cancer-specific mortality (BCSM). Follow-up time was calculated from date of surgery to recurrence event or last follow-up.

Statistical analyses were conducted using SPSS version 30 (Armonk, NY). Baseline clinicopathologic characteristics of the MIBC and unifocal breast cancer cohorts were compared using* t*-tests and chi-squared testing as appropriate. Kaplan–Meier curves were used to estimate 3-year oncologic outcomes of LR, DR, and BCSM. Descriptive statistics were also used to describe a subset analysis of the patients with MIBC who underwent single versus multisite lumpectomies, with chi-squared testing for comparisons. All statistical comparisons were two-sided and performed with a significance level set at *p* < 0.05.

## Results

**Fig. 1:**
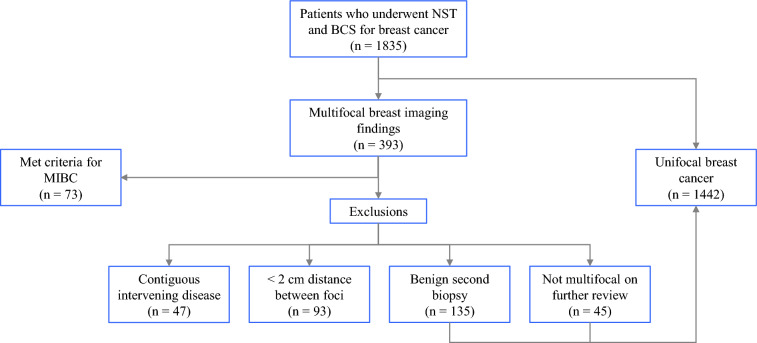
Patient selection criteria; *NST* neoadjuvant systemic therapy, *BCS* breast conserving surgery, *MIBC* multiple ipsilateral breast cancer

From 2016 to 2023, 1515 patients received NST followed by BCS; 73 (4.8%) patients met the eligibility criteria for MIBC and 1442 (95.2%) had unifocal disease. Figure 1 shows the patient selection process, including the application of exclusion criteria, and the resulting final study cohorts. The baseline clinicopathologic characteristics of the cohorts are presented in Table 1. Clinical and pathological stage, tumor grade, and molecular subtype were similarly distributed between the MIBC and unifocal groups. Mixed tumor histology with IDC and ILC was more prevalent among patients with MIBC (17.8% vs. 7.5% in the unifocal cohort, *p* = 0.01). The majority of patients in both cohorts received NAC (82.2% in the MIBC cohort and 83.4% in the unifocal cohort, *p* = 0.78). The remaining patients, all of whom had hormone receptor-positive, HER2-negative (HR+/HER2−) disease received NET. Pathologic complete response (pCR) in the breast occurred in 34.1% of patients overall (27.4% MIBC and 34.5% unifocal breast cancer, *p* = 0.16).

**Table 1:** Baseline clinicopathologic characteristics stratified by tumor focality

Characteristic	Total *N* = 1515 (%)	MIBC *N* = 73 (%)	Unifocal *N* = 1442 (%)	*p*-value
Age				
Median	55 (21–89)	56 (26–84)	55 (21–89)	0.77
< 40	195 (12.9)	12 (16.4)	183 (12.7)	0.13
40–49	321 (21.2)	8 (11.0)	313 (21.7)	
50–59	419 (27.7)	27 (37.0)	392 (27.2)	
60–69	345 (22.8)	16 (21.9)	330 (22.8)	
≥ 70	235 (15.5)	10 (13.7)	225 (15.6)	
Race				
Caucasian	1243 (82.0)	63 (86.3)	1180 (81.8)	0.29
African American	114 (7.5)	6 (8.2)	108 (7.5)	
Asian or Pacific Islander	70 (4.6)	0 (0.0)	70 (4.9)	
Other/Unknown	88 (5.8)	4 (5.5)	84 (5.8)	
Ethnicity				
Not Hispanic	1394 (92.0)	63 (86.3)	1331 (92.3)	0.08
Hispanic	94 (6.2)	9 (12.3)	85 (5.9)	
Unknown	27 (1.8)	1 (1.4)	26 (1.8)	
Type of NST				
NAC	1252 (82.6)	58 (79.5)	1194 (82.8)	0.46
NET	263 (17.4)	15 (20.5)	248 (17.2)	
Tumor histology				
IDC	1259 (83.1)	56 (76.7)	1203 (83.4)	0.01
ILC	99 (6.5)	3 (4.1)	96 (6.7)	
Mixed IDC/ILC	121 (8.0)	13 (17.8)	108 (7.5)	
Other	36 (2.4)	1 (1.4)	35 (2.4)	
Clinical stage				
1	154 (10.2)	10 (13.7)	144 (10.0)	0.58
2	1243 (82.0)	58 (79.5)	1185 (82.2)	
3	118 (7.8)	5 (6.8)	113 (7.8)	
Clinical T				
cT1	316 (20.9)	20 (27.4)	296 (20.5)	0.51
cT2	1062 (70.1)	48 (65.8)	1014 (70.3)	
cT3	133 (8.8)	5 (6.8)	128 (8.9)	
cT4	4 (0.3)	0 (0.0)	4 (0.3)	
Clinical N				
cN0	889 (58.7)	41 (56.2)	848 (58.8)	0.44
cN1	574 (37.9)	31 (42.5)	543 (37.7)	
cN2	39 (2.6)	0 (0.0)	39 (2.7)	
cN3	13 (0.9)	1 (1.4)	12 (0.8)	
Pathological stage				
0	517 (34.1)	20 (27.4)	497 (34.5)	0.16
1	451 (29.8)	19 (26.0)	432 (30.0)	
2	412 (27.2)	23 (31.5)	389 (27.0)	
3	135 (8.9)	11 (15.1)	124 (8.6)	
Pathological T				
pT0	543 (35.8)	21 (28.8)	522 (36.2)	0.30
pT1	607 (40.1)	29 (39.7)	578 (40.1)	
pT2	318 (21.0)	22 (30.1)	296 (20.5)	
pT3	45 (3.0)	1 (1.4)	44 (3.1)	
pT4	2 (0.1)	0 (0.0)	2 (0.1)	
Pathological N				
pN0	1083 (71.5)	47 (64.4)	1036 (71.8)	0.12
pN1	312 (20.6)	15 (20.5)	297 (20.6)	
pN2	92 (6.1)	9 (12.3)	83 (5.8)	
pN3	28 (1.8)	2 (2.7)	26 (1.8)	
Grade				
1	86 (5.7)	6 (8.2)	80 (5.5)	0.61
2	460 (30.4)	25 (34.2)	435 (30.2)	
3	967 (63.8)	42 (57.5)	925 (64.1)	
Unknown	2 (0.1)	0 (0.0)	2 (0.1)	
Molecular subtype				
HR+/HER2−	557 (36.8)	27 (37.0)	530 (36.8)	0.97
HR−/HER2−	432 (28.5)	20 (27.4)	412 (28.6)	
HER2+	526 (34.7)	26 (35.6)	500 (34.7)	
Preoperative MRI				
Yes	898 (59.3)	46 (63.0)	852 (59.1)	0.51
No	617 (40.7)	27 (37.0)	590 (40.9)	
Re-excisions				
0	1316 (86.9)	58 (79.5)	1258 (87.2)	0.15
1	181 (11.9)	14 (19.2)	167 (11.6)	
2	18 (1.2)	1 (1.4)	17 (1.2)	
Final margin status				
> 2 mm	1237 (81.7)	56 (76.7)	1181 (81.9)	0.19
> 0 and ≤ 2 mm	255 (16.8)	17 (23.3)	238 (16.5)	
Positive (tumor on ink)	23 (1.5)	0 (0)	23 (1.6)	
Radiation therapy				
Yes	1392 (91.9)	71 (97.3)	1322 (91.7)	0.20
No	123 (8.1)	2 (2.7)	120 (8.3)	

*IDC* invasive ductal carcinoma, *ILC* invasive lobular carcinoma, *HER2* human epidermal growth factor receptor 2, *HR* hormone receptor, *MIBC* multiple ipsilateral breast cancer, *MRI* magnetic resonance imaging, *NAC* neoadjuvant chemotherapy, *NET* neoadjuvant endocrine therapy, *NST* neoadjuvant systemic therapy

There was no difference in preoperative MRI utilization between cohorts, with 59.3% of patients having MRI. The re-excision rate was 20.6% in the MIBC cohort, with only 1 (1.4%) patient undergoing two re-excisions, and 12.8% in the unifocal breast cancer cohort, with 17 (1.2%) patients undergoing two re-excisions (*p* = 0.15). Final margins were negative in all patients in the MIBC cohort (76.7% > 2 mm and 23.3% between 0 and 2 mm) and in 98.4% of patients in the unifocal cohort (81.9% > 2 mm and 16.5% between 0 and 2 mm, *p* = 0.19).

Among patients in the MIBC cohort, 63 (86.3%) had two sites of disease and 10 (13.7%) had three sites of disease. Among those with two sites of disease, 66 (90.4%) patients had invasive disease as the first and second site while 7 (9.6%) had DCIS as the second site. Of the ten patients with three sites of disease, six (60.0%) had three sites of invasive disease and four (40.0%) had two sites of invasive disease and one site of DCIS. In terms of surgical approach, 48 (65.8%) patients underwent single-site lumpectomy, 23 (31.5%) two-site lumpectomy, and 2 (2.7%) three-site lumpectomy. Baseline patient and tumor characteristics were similar when stratified by surgical approach and are outlined in Table 2. There was a trend toward higher re-excision rates in multisite lumpectomies, but this difference was not statistically significant (14.6% vs. 32.0%, *p* = 0.13). All patients who received NST were treated with standard regimens or on a clinical trial protocol with standard adjuvant therapies. Details are outlined in Supplementary Table 1. Two patients in the MIBC cohort did not have radiation therapy per patient preference; both were node-negative stage IA patients who underwent NET (aged 63 and 75 years).

**Table 2:** Baseline clinicopathologic characteristics and re-excision rates, stratified by surgical approach

Characteristic	Overall *N* = 73 (%)	Single site lumpectomy *N* = 48 (%)	Multiple site lumpectomy *N* = 25 (%)	*p*-Value
Age				
< 40	12 (16.4)	7 (14.6)	5 (20.0)	0.17
40–49	8 (11.0)	6 (12.5)	2 (8.0)	
50–59	27 (37.0)	20 (41.7)	7 (28.0)	
60–69	16 (21.9)	7 (14.6)	9 (36.0)	
≥ 70	10 (13.7)	8 (16.7)	2 (8.0)	
Tumor histology				
IDC	56 (76.7)	35 (72.9)	21 (84.0)	0.68
ILC	3 (4.1)	2 (4.2)	1 (4.0)	
Mixed IDC/ILC	13 (17.8)	10 (20.8)	3 (12.0)	
Other	1 (1.4)	1 (2.1)	0 (0.0)	
Clinical stage				
1	10 (13.7)	4 (8.3)	6 (24.0)	0.06
2	58 (79.5)	42 (87.5)	16 (64.0)	
3	5 (6.8)	2 (4.2)	3 (12.0)	
Pathological stage				
0	20 (27.4)	17 (35.4)	3 (12.0)	0.20
1	19 (26.0)	11 (22.9)	8 (32.0)	
2	23 (31.5)	13 (27.1)	10 (40.0)	
3	11 (15.1)	7 (14.6)	4 (16.0)	
Grade				
1	6 (8.2)	4 (8.3)	2 (8.0)	0.75
2	25 (34.2)	15 (31.3)	10 (40.0)	
3	42 (57.5)	29 (60.4)	13 (52.0)	
Molecular subtype				
HR+/HER2−	27 (37.0)	17 (35.4)	10 (40.0)	0.88
HR−/HER2−	20 (27.4)	14 (29.2)	6 (24.0)	
HER2+	26 (35.6)	17 (35.4)	9 (36.0)	
Preoperative MRI				
Yes	46 (63.0)	29 (60.4)	17 (68.0)	0.52
No	27 (37.0)	19 (39.6)	8 (32.0)	
Re-excisions				
0	58 (79.5)	41 (85.4)	17 (68.0)	0.13
1	14 (19.2)	7 (14.6)	7 (28.0)	
2	1 (1.4)	0 (0.0)	1 (4.0)	
Final margin status				
> 2 mm	56 (76.7)	37 (77.1)	19 (76.0)	0.92
> 0 and ≤ 2 mm	17 (24.0)	11 (22.9)	6 (24.0)	
Positive (tumor on ink)	0	0	0	

*IDC* invasive ductal carcinoma, *ILC* invasive lobular carcinoma, *HER2* human epidermal growth factor receptor 2, *HR* hormone receptor, *MRI* magnetic resonance imaging

At median follow-up of 34.7 months (range 0.8–128 months), LR occurred in 1 (1.4%) patient in the MIBC cohort and 45 (3.1%) patients in the unifocal cohort (*p* = 0.40). The single patient with a local recurrence in the MIBC cohort had clinical T2N1 high-grade triple negative IDC at two sites and underwent preoperative MRI during workup. Following neoadjuvant dose-dense doxorubicin and cyclophosphamide followed by paclitaxel, BCS was performed as a single-site lumpectomy and final pathology revealed a 0.05 cm focus of residual microinvasive ductal carcinoma with associated high-grade DCIS and one negative sentinel lymph node; all margins were > 2 mm. The patient then completed whole-breast RT with high tangents. The LR presented as an irregular mass at the lumpectomy site and occurred at 1 year after completion of initial surgery. In the unifocal cohort, MRI use (23/45 patients with LR did not have MRI and 22/45 patients had MRI, *p* = 0.16) and positive margins (2/21 [4.4%] patients with positive margins and 43/1376 [3.0%] patients with negative margins had LR *p* = 0.12) were not associated with LR.

There was no difference in DR or BCSM rates between groups (Table 3). In the MIBC and unifocal breast cancer cohorts, DR was identified in 4 (5.5%) and 66 (4.6%) patients and BCSM occurred in 3 (4.1%) and 38 (2.6%) patients, respectively. The 3-year unadjusted Kaplan–Meier estimates of LR, DR, and BCSM also showed no statistically significant differences between the MIBC and unifocal groups.

**Table 3:** Outcomes for MIBC versus unifocal breast cancer cohorts

Outcome	MIBC *N* = 73 (%)	Unifocal breast cancer *N* = 1442 (%)	*p*-Value
Overall incidence			
LR	1 (1.4)	45 (3.1)	0.40
DR	4 (5.5)	66 (4.6)	0.37
BCSM	3 (4.1)	38 (2.6)	0.45
3-year Kaplan–Meier estimates			
3-year LR	1.6%	1.8%	0.30
3-year DR	7.0%	3.2%	0.97
3-year BCSM	5.1%	2.3%	0.30

*MIBC* multiple ipsilateral breast cancer, *LR* local recurrence, *DR* distant recurrence, *BCSM* breast-cancer-specific mortality

## Discussion

Prior to the ACOSOG Z11102 trial, acceptance of BCS for MIBC was inconsistent due to concerns about higher LR rates with multiple disease sites and a lack of support from consensus guidelines.^9^ Contemporary large retrospective studies of patients with MIBC undergoing BCS have since shown more acceptable rates of LR with no significant difference in disease-free survival (DFS) and overall survival (OS) when compared with patients with unifocal disease.^10,11^ A systematic review of the literature in 2018 found equivalent rates of locoregional recurrence (LRR) for MIBC treated with upfront BCS or mastectomy.^12^ The ACOSOG Z11102 trial was the first prospective study to assess breast-conservation therapy (BCT) for MIBC and provided evidence to support the safety of this approach. These findings led to its incorporation into updated consensus guidelines, with the 2023 St. Gallen recommendations marking the first endorsement of BCT for MIBC.^13^

Although BCS for MIBC in upfront surgery has been incorporated into clinical guidelines, there are limited data on the safety of this approach after NST. While contemporary series have shown the safety of BCT after NST for unifocal disease,^14–16^ theoretical concerns remain in the setting of MIBC. These include concerns about leaving behind small amounts of systemic-therapy-resistant disease outside the margins of resection and not resecting the full tumor treatment bed when disease has responded. Further, optimal margin width has not been as clearly defined after NST due to different patterns of treatment response, although several retrospective series support no tumor on ink as a negative margin in the NST setting.^17–19^ In this study, we found that patients with MIBC selected for BCS after NST have equivalent 3-year oncologic outcomes as a cohort of patients with unifocal breast cancer over the same timeframe, suggesting this is a feasible option in well-selected patients.

Use of NST comes with a local therapy benefit of increasing eligibility for BCS in patients who were previously borderline candidates or not eligible for this approach.^20^ Patients with MIBC are therefore a specific group of interest in whom NST may enable surgical downstaging and increase eligibility for BCT. To date, few studies have specifically addressed the oncologic safety of BCS in patients with MIBC after NST. A retrospective study from the University of Texas MD Anderson Cancer Center examined 97 patients with MIBC treated with NAC followed by locoregional therapy from 1976 to 2003.^11^ Only 20/97 (20.6%) patients with multifocal or multicentric breast cancer underwent BCT, and when compared with patients with unifocal tumors treated during the same timeframe, there was no significant difference in 5-year local recurrence-free survival (LRFS) or LRR (7% vs. 10%, *p* = 0.78). A post hoc analysis of data from a series of clinical trials conducted in Germany was used to evaluate the impact of tumor focality on type of surgery, LRFS, DFS, and OS.^21^ Of 6134 patients who received NAC, 1401 had multiple tumor foci and 44.0% underwent BCT, with a 3-year LRR of 5.2% and no significant difference in LRFS, DFS, or OS in patients with multifocal or multicentric breast cancer who underwent BCT when compared with a unifocal tumor group. More recently, a retrospective cohort study from two institutions in Canada evaluated 106 patients with MIBC undergoing BCS post-NAC and similarly found no difference in LRFS, RFS, or OS compared with patients with unifocal disease.^22^ This study reported a LR rate of 4.7% at 4.6 years of follow-up and found that residual disease, close or positive margins, and omission of RT were associated with increased odds of LR. Our study of 73 patients meeting clearly defined criteria for MIBC and undergoing BCS after NST adds to the growing body of literature that with appropriate patient selection and adherence to consistent oncologic principles, LR rates and survival outcomes are similar to patients receiving treatment for unifocal disease.

Patients recommended for NAC often have higher risk disease subtypes, and the risk of distant disease events in these patients outnumbers the risk of local events, a finding which was also true in our patient cohorts.^23–25^ When compared with the 5-year DR rate of 3.6% in the Alliance Z11102 trial, we report a higher rate of DR in patients with MIBC, which can be attributed to the higher risk disease presentation in our NST-treated population, with the majority of patients with MIBC presenting with cT2/T3 and clinically node-positive disease and about two-thirds with HER2+ or triple-negative breast cancer. Despite these higher-risk characteristics among patients with MIBC undergoing NST, DR and BCSM remained similar between our two patient cohorts. Furthermore, 3-year unadjusted Kaplan–Meier estimates of LR, DR, and BCSM were not different, further suggesting the safety of BCS in this population.

Patients with MIBC who receive NET represent a distinct population from those treated with NAC, as NET is typically offered to postmenopausal patients with strongly HR+/HER2−, low-grade tumors that are more indolent in nature.^26^ Several studies have shown that NET is equivalent in downstaging tumors for BCS compared with NAC in HR+/HER2− breast cancer, although pCR is rare.^26–28^ This rationale also applies to patients with MIBC, and NET can likely be used as a safe option for downstaging tumors to BCT with more acceptable cosmetic outcomes. Our study included 13 (17.8%) patients in the MIBC cohort who received NET with no incidence of LR during the study follow-up period of 34.7 months. Although this is short follow-up for HR+/HER2− recurrences, which can occur decades after initial treatment, this finding supports the feasibility of this approach for downstaging tumor burden to achieve BCS.

Imaging plays a key role in surgical planning for patients with MIBC, particularly for those undergoing NST with the goal of breast conservation. In the neoadjuvant setting, preoperative MRI has been widely used to assess treatment response and eligibility for BCS.^29,30^ In our MIBC cohort, the overall rate of preoperative MRI was 63.0%. Some patients were diagnosed with MIBC on the basis of preoperative MRI, while others had MIBC identified on mammography and ultrasound alone, which may reflect differences in tumor burden or extent of disease. The Z11102 trial reported a higher rate of LR at 5 years among those without preoperative MRI, 22.6% versus 1.7% in those with preoperative MRI (*p* = 0.002); however, this analysis was limited, as only 15 patients without MRI were enrolled in the trial.^8^ Notably, MRI identified an additional malignant focus not seen on mammogram or ultrasound in 42.1% of trial participants. Thus, we would advocate for routine preoperative MRI use in patients with MIBC being considered for BCS after NST.

Surgical approaches for BCS in patients with MIBC include single-site and multisite lumpectomies. Among the patients with MIBC in our study, 65.8% underwent single-site lumpectomy and 79.5% did not require re-excision, a rate comparable to that of the unifocal BC cohort. A key consideration is the impact of multiple lumpectomy sites on radiation planning, including whole-breast dose distribution and boost volume, which may affect long-term cosmetic outcomes given the potential for increased fibrosis or contour deformities. The Alliance Z11102 trial reported that 67.6% of patients achieved negative margins on the initial excision, and the majority of patients reported good to excellent cosmetic outcomes.^8^ These findings highlight the importance of multidisciplinary discussion and coordinated planning between surgery and radiation oncology for patients with MIBC treated with NST to optimize oncologic and cosmetic outcomes.

Our study has several limitations. It was a retrospective analysis from a single institution, and thus there is inherent selection bias for which patients were selected for BCT following NST. However, we used a strict definition of MIBC based on preoperative imaging criteria. Our results therefore demonstrate the importance of careful patient selection. There was a limited sample size for the MIBC cohort resulting in a low number of events, which restricts the ability to perform further analyses of oncologic outcomes. In addition, details regarding adjuvant treatments were variably documented, particularly with respect to type of radiation therapy if received at outside institutions. The relatively short follow-up also limits evaluation of long-term oncologic outcomes, particularly for patients with HR+/HER2− disease.

## Conclusions

In this study of patients with MIBC selected for BCS after NST, LR was 1.4% at a median follow-up of 3 years, and other short-term oncologic outcomes were similar to patients with unifocal breast cancer undergoing BCS after NST. These findings suggest that BCS is a safe surgical option for well-selected patients with MIBC undergoing NST.

## Supplementary Information

Below is the link to the electronic supplementary material.

Supplementary file1 (DOCX 14 KB)

## Data Availability

The data that support the findings of this study are available from the corresponding author upon reasonable request.
