# Supplementary material for: Breast-Conserving Therapy for Multiple Ipsilateral Breast Cancer After Neoadjuvant Systemic Therapy
Source: Ann Surg Oncol. 2026 Jan 3;33(4):3355–63. doi: 10.1245/s10434-025-18939-6 (PMC12982253; doi:10.1245/s10434-025-18939-6)
Supplement: Supplementary file 1 — Supplementary file1 (DOCX 14 KB) [file 10434_2025_18939_MOESM1_ESM.docx]

**Supplemental Table 1. Systemic Therapy Details in MIBC Cohort (n=73)**

| Treatment Details | NAC (n=58) | NET (n=15) |
| --- | --- | --- |
| **Neoadjuvant Systemic Therapy** |  |  |
| Doxorubicin + cyclophosphamide+ paclitaxel (ACT)  Doxorubicin + cyclophosphamide (AC)  Doxorubicin + Cyclophosphamide + Paclitaxel + Carboplatin + Pembrolizumab  Doxorubicin/Cyclophosphamide/Paclitaxel/Trastuzumab/Pertuzumab (AC-THP)  Docetaxel/Carboplatin/Trastuzumab/Pertuzumab (TC-HP)  Paclitaxel/Trastuzumab/Pertuzumab (THP)  Sacituzumab govitecan  TDM-1  Docetaxel/ Cyclophosphamide (TC)  Cyclophosphamide/Methotrexate/Fluorouracil (CMF)  Aromatase Inhibitor  Aromatase Inhibitor + CDK4/6 Inhibitor | 18  3  5  4  2  20  1  1  3  1 | 13  2 |
| **Adjuvant Therapy** |  |  |
| Capecitabine  Carboplatin  Doxorubicin/Cyclophosphamide/Paclitaxel (ACT)  Doxorubicin/Cyclophosphamide/T-DM1  Doxorubicin/Cyclophosphamide/Trastuzumab (AC/Trastuzumab)  Paclitaxel  Paclitaxel/Trastuzumab/Pertuzumab (THP)  Other  Pembrolizumab  Pembrolizumab/Capecitabine  TDM1  Trastuzumab  Trastuzumab/Pertuzumab (HP)  Aromatase Inhibitor  Tamoxifen | 6  1  1  4  1  2  1  2  3  2  6  4  9  23  7 | 2  13  1 |
